# Mutational monitoring of *EGFR* T790M in cfDNA for clinical outcome prediction in *EGFR*-mutant lung adenocarcinoma

**DOI:** 10.1371/journal.pone.0207001

**Published:** 2018-11-16

**Authors:** Kang-Yi Su, Jeng-Sen Tseng, Keng-Mao Liao, Tsung-Ying Yang, Kun-Chieh Chen, Kuo-Hsuan Hsu, Pan-Chyr Yang, Sung-Liang Yu, Gee-Chen Chang

**Affiliations:** 1 Department of Clinical Laboratory Sciences and Medical Biotechnology, College of Medicine, National Taiwan University, Taipei, Taiwan; 2 Department of Laboratory Medicine, National Taiwan University Hospital, Taipei, Taiwan; 3 Center of Genomic Medicine, National Taiwan University, Taipei, Taiwan; 4 Genome and systems Biology Degree Program, National Taiwan University and Academia Sinica, Taipei, Taiwan; 5 Division of Chest Medicine, Department of Internal Medicine, Taichung Veterans General Hospital, Taichung, Taiwan; 6 Faculty of Medicine, School of Medicine, National Yang-Ming University, Taipei, Taiwan; 7 Department of Internal Medicine, College of Medicine, National Taiwan University, Taipei, Taiwan; 8 Department of Pathology and Graduate Institute of Pathology, College of Medicine, National Taiwan University, Taipei, Taiwan; 9 Center for Optoelectronic Biomedicine, College of Medicine, National Taiwan University, Taipei, Taiwan; 10 Graduate Institute of Clinical Medicine, National Taiwan University College of Medicine, Taipei, Taiwan; 11 Institute of Biomedical Sciences, National Chung Hsing University, Taichung, Taiwan; University of South Alabama Mitchell Cancer Institute, UNITED STATES

## Abstract

Several ultra-sensitive methods for T790M in plasma cell-free DNA (cfDNA) have been developed for lung cancer. The correlation between mutation-allele frequency (MAF) cut-off, drug responsiveness, and outcome prediction is an unmet needs and not fully addressed. An innovative combination of peptide nucleic acid (PNA) and Matrix-Assisted Laser Desorption/Ionization Time-of-Flight Mass Spectrometry (MALDI-TOF MS) was used to proof of concept for monitoring cfDNA T790M in *EGFR*-mutant patients. Mutant enrichment by PNA was optimized and the detection limit was evaluated through serial dilutions. The cut-off value was identified by receiver-operating-characteristic (ROC) curve analysis utilizing serial sampled plasmas of patients from EGFR-tyrosine kinase inhibitor (TKI) pretreatment to progressive-disease (PD). Results, comparisons, and objective response rate (ORR) were analyzed in 103 patients’ tumor and cfDNA T790M, with 20 of them receiving an additional COBAS test. The detection limit was 0.1% MAF. The cut-off for PD and imminent PD was 15% and 5% with an ROC area under the curve (AUC) of 0.96 and 0.82 in 2 ml plasma. Detection sensitivity of cfDNA T790M was 67.4% and overall concordance was 78.6%. ORR was similar in T790M-positive cfDNA (69.6%) and tumor samples (70.6%) treated with osimertinib. Among 65 T790M-positive tumors, 15 were negative in cfDNA (23.1%). Seven of 38 T790M-positive cfDNA samples were negative in the tumors (18.4%). PNA-MALDI-TOF MS had a higher detection rate than COBAS. In conclusion, identification of T790M cut-off value in cfDNA improves cancer managements. We provide a strategy for optimizing testing utility, flexibility, quality, and cost in the clinical practice.

## Introduction

Patients with mutant EGFR-driven lung adenocarcinoma receiving EGFR tyrosine kinase inhibitor (TKI) therapy had better response rates and progression-free survival (PFS) when compared to those receiving traditional standard chemotherapy [1]. However, most patients will eventually develop drug resistance, with a median PFS of around 9 to 13 months. In 50% to 60% of relapse cases, *EGFR* T790M is the major cause of resistance [2, 3]. Third-generation EGFR-TKIs, such as osimertinib, rociletinib, HM61713/BI1482694, ASP8273, and EGF816, which can specifically target *EGFR* T790M, have objective response rates (ORRs) of between 45% and 70% [4–6], and a median PFS of around 10 months [4, 5, 7, 8].

The regulatory approval of osimertinib contributed to the emergence of *EGFR* T790M testing as a companion diagnostic. However, since genetic testing-based therapy was put into the guidelines for treating advanced lung adenocarcinoma, the challenge lies in the availability of rebiopsy tissues from patients who failed to respond to treatment with first or second-generation EGFR-TKIs. Therefore, a liquid biopsy such as cell-free plasma DNA (cfDNA) is used for molecular testing with advanced technologies for patients with advanced lung adenocarcinoma following EGFR-TKI treatment failure [9]. Although cfDNA can be fluctuated significantly with multiple factors such as quantity and quality issues [10, 11], cfDNA biopsy has been widely used as a diagnostic strategy to identify targetable genetic mutations with the improvement of testing techniques. It has also been used to monitor disease progression and treatment efficacy [12]. Following approval by the FDA, the COBAS *EGFR* mutation test v2 (Roche) became the new proposed paradigm of treatment guidelines for *EGFR* T790M by testing cfDNA prior to tumor tissue, and has been applied in clinical practice [13]. In addition, several highly-sensitive and ultra-sensitive methods such as Therascreen (ARMS, Qiagen) have been developed with the aim of better meeting clinical needs. However, several issues are poorly addressed, such as the amount and quality of cfDNA to be sampled, the convenience of multiplex testing, and the costs of testing.

Previously, we developed a nucleotide matrix-assisted laser desorption ionization time-of-flight mass spectrometry (MALDI-TOF MS) assay consisting of 25 multiplex probes to detect over 50 types of four cancer-driving mutations in Taiwan [14]. We implemented this approach in routine molecular diagnostics for more than 8000 cases in an ISO15189-certified central laboratory [15]. Methods with high sensitivity can identify that pretreatment *EGFR* T790M is correlated with treatment outcome [14, 16]. Identifying mutations in cfDNA samples may require ultra-sensitive platforms, however. Improving detection through ultra-sensitive methods creates challenges in the correlation of analytical validation and clinical validation. In other words, the MAF cut-off of *EGFR* T790M and its relation to clinical outcomes should be evaluated. We therefore utilized an ultra-sensitive method combining peptide nucleic acid (PNA) with a MALDI-TOF MS platform (PNA-MALDI-TOF MS) to detect T790M in cfDNA from advanced lung adenocarcinoma patients. Our most important finding was that according to our standardized testing procedure, the MAF cut-off of *EGFR* T790M closely predicts imminent progressive disease (PD) during EGFR-TKI treatment.

## Materials and methods

### Patients and specimens

This study consisted of two advanced *EGFR*-mutant lung adenocarcinoma patient groups diagnosed and treated from May 2014 to Apr 2017 at Taichung Veterans General Hospital (TCVGH). One is a prospective training cohort consisted of five *EGFR*-mutant patients developed *EGFR* T790M in rebiopsy tumors after first-line EGFR-TKIs treatment failure. The inclusion criteria of patients was they had serial plasmas collected before (pretreatment), during (at least 2 times), and after EGFR-TKIs (PD) treatment. All plasmas were used to identify the ideal cut-off MAF of cfDNA T790M. The other is from a retrospective testing cohort contained paired rebiopsy tumors and cfDNA after EGFR-TKIs treatment failure. All were used to evaluate the correlation in *EGFR* T790M detection between tumors and cfDNA. In addition, twenty patients being treated with osimertinib in the testing cohort were selected for comparing COBAS and PNA-MALDI-TOF MS in cfDNA, using tumors as a standard. The outcomes of patients treated with osimertinib were also analyzed.

Clinical data for analysis included patients’ age, gender, smoking status, Eastern Cooperative Oncology Group performance status (ECOG PS), *EGFR*-mutation subtype status, prior EGFR-TKIs treatment, and PFS. Unidimensional measurements as defined by the Response Evaluation Criteria in Solid Tumors (RECIST) version 1.1 were used in this study. The study was approved by the TCVGH institutional review board with approval number: No.C08179. All of the patients provided written informed consent in the procurement of tumor and plasma specimens.

### Plasma DNA extraction

Plasma cfDNA was extracted using the QIAamp Circulating Nucleic Acid Kit (QIAGEN, Hilden, Germany) according to the users’ manual. In brief, 2 ml of EDTA (Ethylenediaminetetraacetic acid) plasma was used as starting material and the final elution volume of the column was 60 μl.

### Mutation detection and biochemical reaction of PNA-MALDI-TOF MS for *EGFR* T790M

Baseline EGFR T790M detection in tumor biopsy was utilized the MALDI-TOF MS platform according to the standard operation procedure in the ISO15189 certified clinical center laboratory based on our previous studies [14, 15]. For EGFR T790M detection in cfDNA, the combination of PNA and MALDI-TOF MS method was utilized according to the method used in tumor biopsy with some modification. Briefly, PNA oligos (5’-GCTCATCACGCAGCTCA-3’) (only for T790M) used to specifically lock wild-type allele from the previous study [17] were synthesized by PanaGene (Daejeon, Korea). The final optimized concentration of PNA to efficiently block wild-type alleles was 25 μM in PCR reactions. All nucleotide mass spectrometry assays were utilized MassARRAY System (Cat. No.10411, SEQUENIM, San Diego, CA acquired by Agena Bioscience, http://agenabio.com/, San Diego, CA at 2014) according to users’ manual. The biochemical reaction was based on the users’ manual. Briefly, a total volume of 5 μl mixture containing 1 μl extracted plasma DNA, 0.5 unit HotStarTaq DNA polymerase, 500 μM dNTPs, 100 nM of T790M locus primers, 25 μM PNA, 1.25 μl of 10x HotStar buffer and additional 1.625 mM MgCl_2_ was subjected to PCR reactions with condition as follows. A single activation cycle at 94°C for 15 min followed by 45 touch-down amplification cycles, consisting of 15 cycles of 94°C for 20 sec, 61°C annealing for 30 sec, 72°C for 60 sec and another 30 cycles with 57°C annealing for 30 sec. The PCR products were then treated with SAP for dNTP neutralization as following: 0.5 unit SAP with 1.7× SAP buffer was prepared into a final of 2 μl mix and then added to the PCR product for 40 min incubation at 37°C followed by 5 min inactivation at 80°C. Next, the SAP-treated PCR products were subjected to the single nucleotide extension reaction by using iPLEX Pro reagent kit containing Sequenase 0.04 μl, termination mix 0.1 μl, 10× iPLEX Pro buffer 0.2 μl and T790M probes with a final concentration of 7 to 14 μM in total of 2 μl mixture. Temperature cycling consisted of a modified 60°C annealing and 200-cycle extension method (94°C, 30 sec followed by 40 repeats of 5 rounds of 94°C for 20 sec, 80°C for 5 sec, 60°C for 5 sec). After desalting with SpectroClean Resin, samples were loaded onto the matrix of SpectroCHIP by MassARRAY Nanodispenser RS1000 then analyzed by Bruker Autoflex MassARRAY Analyzer 4 MALDI-TOF MS. Data were collected and analyzed by MassARRAY Typer (version 4) software (SEQUENOM). The signal presented in the correct mass position (corresponding to products) and passed the criteria of signal to noise ratio with acceptable probability (>0.8) will be interpreted as a positive result by MassARRAY Typer (version 4) software automatically.

### Analytical and clinical MAF cut-off value identification

The analytical cut-off value was followed the previous study [14]. Briefly, it was estimated by *EGFR* T790M mutant expression plasmids (pcDNA3.1-EGFR^L858R/T790M^) serially diluted with *EGFR* wild-type ones (pcDNA-EGFR^WT^). In addition, DNA extracted from health individuals were also utilized to identify cut-off value and background noise. The MAF by MALDI-TOF MS in each sample was calculated as: % = (mutant-type height)/(mutant-type height+wild-type height) x 100 from the average of two independent replications. For clinical cut-off value identification for outcome prediction, receiver operating characteristic (ROC) curve was utilized. The ROC curve of each testing plasma volume was drawn according to *EGFR* T790M MAF from totally 30 serial sampled plasmas of five patients and corresponding clinical outcomes (PD or imminent PD).

## Results

### Patient characteristics and specimen collection

**Fig 1:**
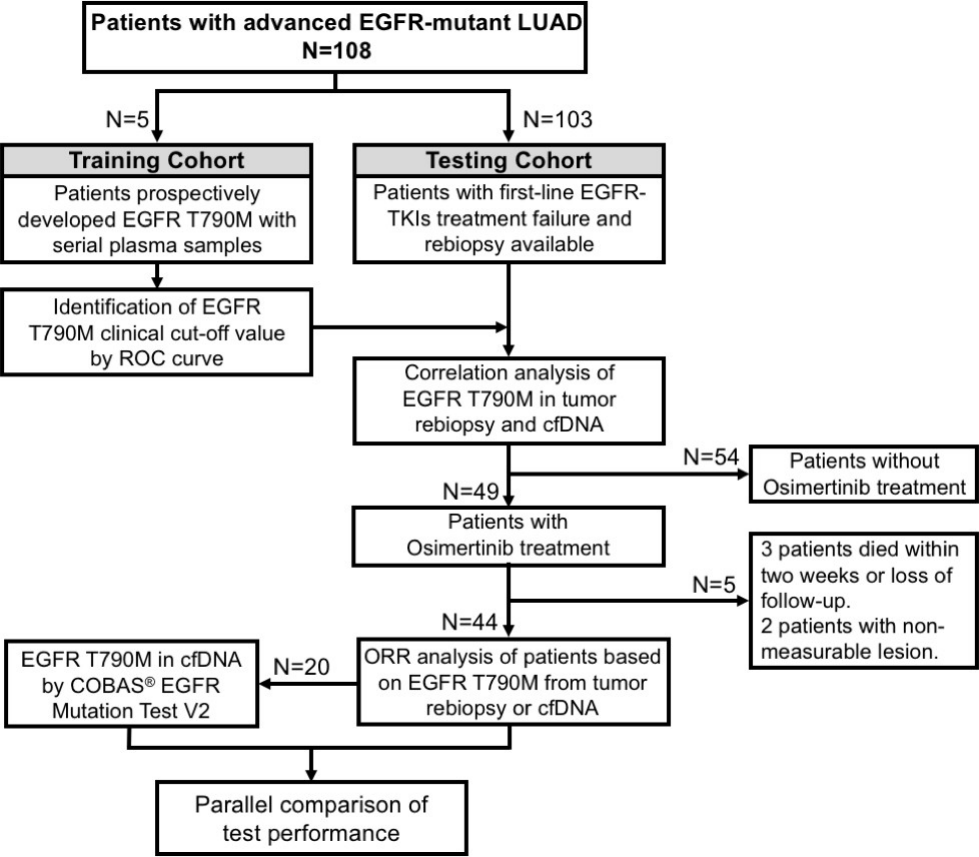
Experimental design and testing flowchart. The study is started from a training cohort for EGFR T790M cut-off value identification and a testing cohort for clinical validation.

The experimental design and testing flowchart were described in Fig 1. Two cohorts including the training cohort for cut-off value identification and the testing cohort for validation were processed independently. The characteristics of training cohort is summarized (Table 1). All of them were *EGFR*-mutant patients and achieved an objective response to first-line EGFR-TKIs treatment. Totally 30 plasmas (2 patients with 7 serial plasmas, 2 patients with 6 serial plasmas, and 1 patient with 4 serial plasmas) with corresponding outcomes were further utilized for cut-off identification by ROC curve.

**Table 1:** Characteristics of 5 patients utilized for identification of cutoff value.

No.	Age	Gender	Smoking status	Baseline *EGFR* status	EGFR-TKI	Best response	PFS (m)
PR232	48	Female	NS	19Del	Gefitinib	PR	14.5
PR251	46	Female	NS	L858R	Gefitinib	PR	16.3
PR306	50	Male	NS	L858R	Erlotinib	PR	10.6
PR353	60	Female	NS	L858R	Erlotinib	PR	16.8
PR366	39	Female	NS	L858R	Erlotinib	PR	11.9

EGFR, epidermal growth factor receptor; TKI, tyrosine kinase inhibitor; PFS, progression-free survival; NS, non-smokers; PR, partial response.

The testing cohort of 103 patients were all advanced *EGFR*-mutant lung adenocarcinoma patients, including 95 (92.2%) with acquired resistance to EGFR-TKIs and 8 (7.8%) with de novo T790M. Their ages ranged from 32–89 years old, with a median age of 60. Sixty-three (61.2%) were female and 83 (80.6%) were non-smokers. Baseline *EGFR*-mutations were identified by MALDI-TOF MS according to previous studies [14, 15]. They included 54 (52.4%) with an exon19 deletion (del19), 35 (34.0%) with L858R, and 14 (13.6%) with other mutations (One G719S, one del19+G719C, five del19+T790M, one L858R+E709G, three L858R+T790M, and three L861Q). Forty-six (44.7%) received osimertinib treatment (Table 2). The median age was 61. Thirty-two (69.6%) were female and 38 (82.6%) were non-smokers. Exon19 deletion was the most common of the *EGFR* mutation types (58.7%). Thirty-seven (80.4%) received EGFR-TKIs as their first line of therapy and 37 (80.4%) received at least one chemotherapy regimen. Thirty-five (76.1%) had ECOG PS 0–1 at the time of osimertinib treatment. After exclusion of 2 patients without measurable lesions, the ORR and DCR (disease control rate) of osimertinib treatment were 61.4% and 86.4%, respectively. The median PFS was 9.6 months (95% CI 6.8–12.4).

**Table 2:** Demographic data of patients treated with osimertinib (n = 46).

Characteristics	n = 46
Age, median (range) (yrs)	61 (43–90)
Gender, n (%)	
Male	14 (30.4)
Female	32 (69.6)
Smoking status, n (%)	
Non-smokers	38 (82.6)
Former and current smokers	8 (17.4)
Baseline *EGFR* mutations, n (%)	
Exon 19 deletions	27 (58.7)
Exon 21 L858R	10 (21.7)
Others^1^	9 (19.6)
First EGFR-TKIs regimen, n (%)	
Gefitinib	23 (50.0)
Erlotinib	18 (39.1)
Afatinib	4 (8.7)
N/A^2^	1 (2.2)
Initial EGFR-TKIs treatment, n (%)	
First line	37 (80.4)
Second line or later	8 (17.4)
N/A^2^	1 (2.2)
Prior EGFR-TKI(s) treatment, n (%)	
0	1 (2.2)
1	27 (58.7)
2 or 3	18 (39.1)
Prior chemotherapy(ies)	
0	9 (19.6)
1	16 (34.8)
2 or more	21 (45.7)
ECOG PS, n (%)	
0–1	35 (76.1)
2 or more	11 (23.9)

EGFR, epidermal growth factor receptor; TKIs, tyrosine kinase inhibitors; N/A, not applicable; ECOG PS, Eastern Cooperative Oncology Group performance status.

^1^Include complex mutations involving 19Del or L858R.

^2^One patient harboring primary T790M did not receive EGFR-TKIs before osimertinib.

### Optimization of PNA-MALDI-TOF MS for *EGFR* T790M detection

**Fig 2:**
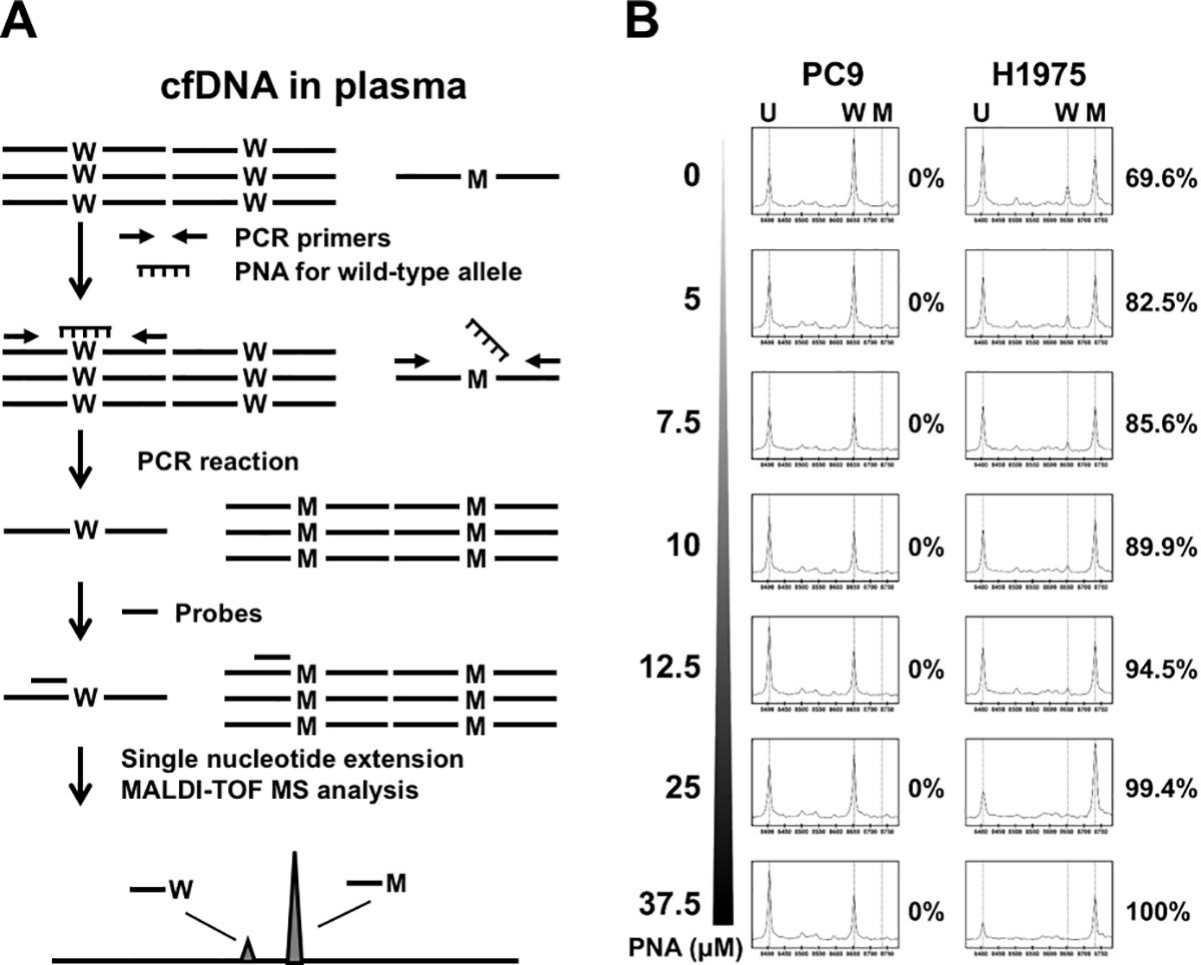
PNA-MALD-TOF MS mutation detection and PNA concentration optimization. (A) Detection principles of PNA-MALDI-TOF MS. The PNA for the corresponding wild-type allele is added with PCR primers for detection locus amplification. The mutant allele, but not the wild-type allele, is amplified after PCR, followed by single nucleotide extension. The single nucleotide extension is performed by probes specifically annealed to the nucleotide before the mutant site. The final product, with different mass due to wild-type/mutant incorporated nucleotides, was analyzed by MALDI-TOF MS. (B) Evaluation of wild-type allele inhibition efficacy. PC9 cells without *EGFR* T790M and H1975 cells with *EGFR* T790M were used for pilot testing. The mutation frequency (mutant alleles/(mutant+wild-type alleles)x100%) of *EGFR* T790M was 0% and 69.6% in PC9 and H1975 cells, respectively. The mutation frequency of *EGFR* T790M in H1975 cells was proportionally elevated with increasing concentrations of PNA. U, unextend probe; W, wild-type signal; M, mutation (*EGFR* T790M) signal.

The overall concept of *EGFR* T790M detection in cfDNA by nucleotide MALTI-TOF MS with PNA is illustrated in Fig 2A. cfDNA is assessed using PCR with additional *EGFR*-Thr790 wild-type allele PNA for amplification inhibition followed by MassARRAY analysis according to our previous studies [14, 15, 18]. In order to evaluate the efficacy of wild-type allele inhibition, the concentration of PNA should be optimized before testing. H1975 cells harboring L858R/T790M mutations and PC9 cells without *EGFR* T790M were utilized for pilot testing (Fig 2B). In the absence of *EGFR* T790M PNA, the MAF of *EGFR* T790M in H1975 cells was 69.6%, similar to our previous study [15]. With the increase in PNA concentration from 5 μM (1x) to 37.5 μM (7.5x), the MAF of *EGFR* T790M rose due to the inhibition of wild-type alleles (Fig 2B). However, *EGFR* T790M PNA did not affect del19 in PC9 cells (Fig 2B). Considering inhibition efficacy, PNA interference, and consumption of PNA, we determined 25 μM as a final concentration for further testing.

### Evaluation of detection limitation and stability

**Fig 3:**
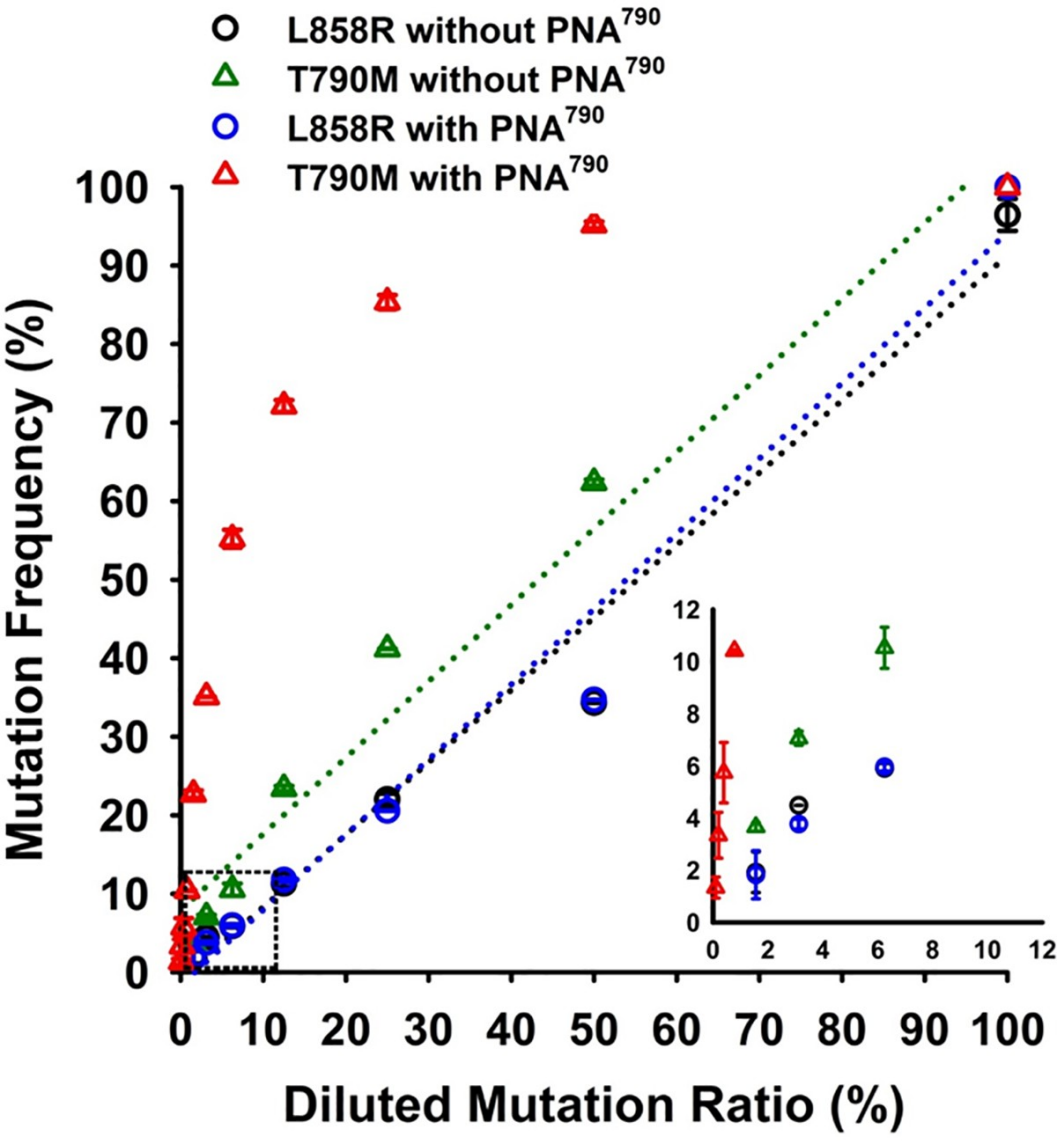
Evaluation of *EGFR* T790M detection limitation by PNA-MALDI-TOF MS. Wild-type (pcDNA3.1-EGFR^wild-type^) and mutant (pcDNA3.1-EGFR^L858R/T790M^) *EGFR* expression plasmids were proportionally mixed to generate serial mutation ratio samples to test the limit of detection. MALDI-TOF MS without *EGFR* T790M PNA had a detection sensitivity approximating 1% both for L858R and T790M. T790M, but not L858R, had a 0.1% detection sensitivity when combined with PNA.

Testing was performed in serial *EGFR* T790M-contained plasmid mixtures with or without PNA (Fig 3). In the absence of any PNA, the MAF of both *EGFR* L858R and T790M by MALDI-TOF MS were highly correlated with the theoretical T790M ratio (Fig 3). In terms of *EGFR* T790M PNA results, mutation frequency of T790M was significantly higher than the theoretical T790M ratio due to mutant enrichment. In the mention of L858R, the MAF was similar with the diluted mutation ratio due to the absence of L858R specific PNA. The limit of detection was around 1% for L858R and T790M in the absence of *EGFR* T790M PNA, and 0.1% for T790M in the presence of *EGFR* T790M PNA. The MAF of T790M in both plasmids without T790M and DNA from healthy individuals was 0%. In the mention of testing precision, within-run repeatability (n = 20) and between-run reproducibility (n = 20) were evaluated by T790M harbored H1975 cells (S1 Fig). The coefficient of variation (CV) of within-run and between-run was 0.57% and 0.55% respectively. In addition to non-clinical validation, we also evaluated the CV, one important indicator of measurement uncertainty, in real patient samples. Plasma samples with low, median, and high T790M MAF (n = 5 for each group) were further tested in triplicates for analysis (S2 Table). The mean CV was 17.9% for low enriched MAF (<5%), 12.9% for median enriched MAF (5–15%), and 3.6% for high enriched MAF (>15%). In conclusion, PNA can improve detection limitation up to 10-fold with high precision and clear results.

### Identification of the mutation frequency cut-off value related to PD and imminent PD

**Fig 4:**
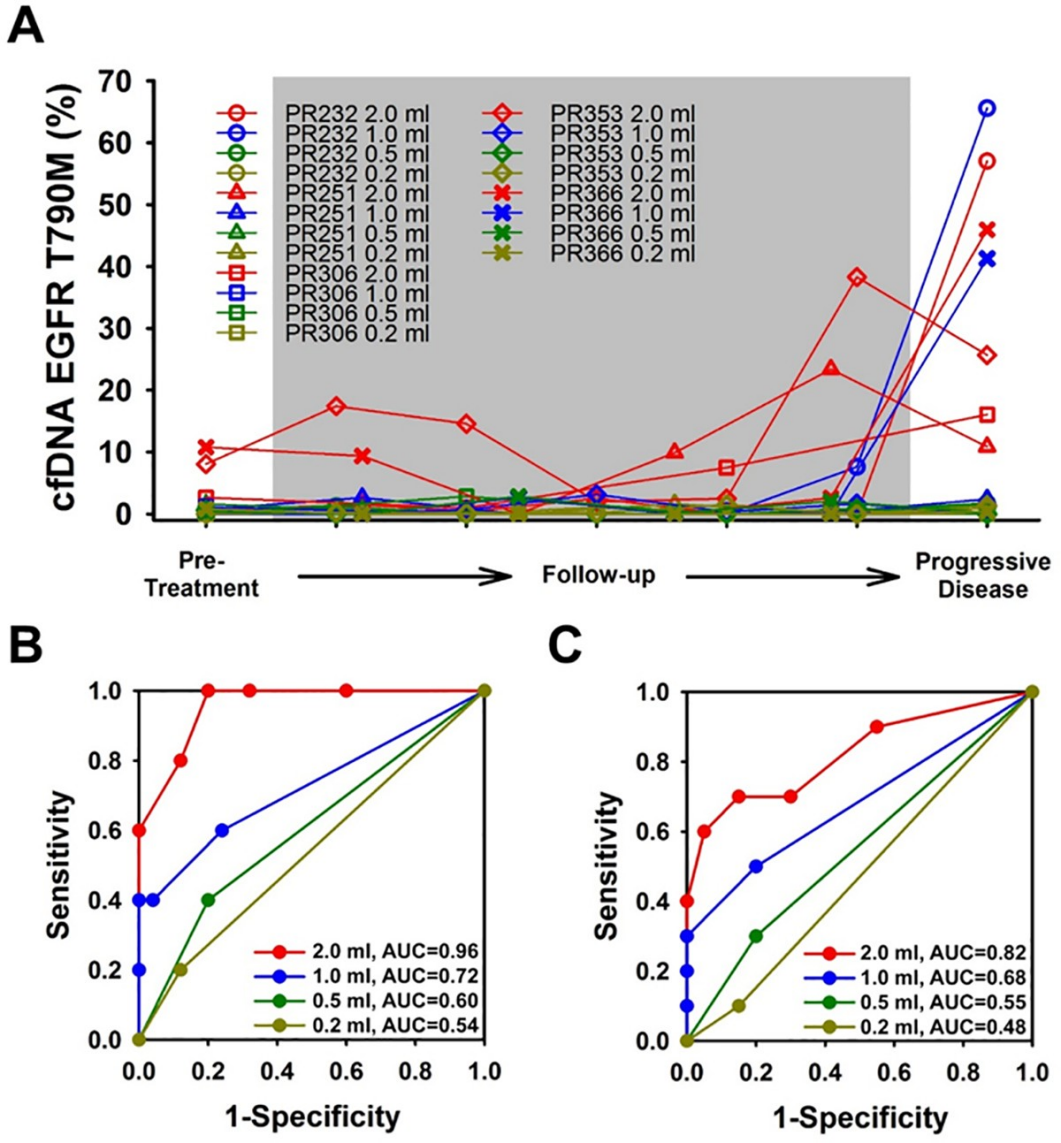
Evaluation of the correlation between *EGFR* T790M mutation frequency and clinical treatment response. (A) Variations of *EGFR* T790M mutation frequency during *EGFR*-TKI treatment. Plasma samples from pretreatment, follow-up, and progressive disease (PD) stages of five patients (PR232, PR251, PR306, PR353, and PR366) were tested for *EGFR* T790M. Different volumes of plasma at each sampling time were used as starting material to evaluate testing efficacy. The sequential mutation frequency of each patient was plotted from pretreatment to PD. (B) Receiver optimizing characteristic (ROC) curve for prediction evaluation, taking the time of PD as the clinical event. (C) ROC curve for prediction evaluation, taking the time of the follow-up stage nearest PD (imminent PD) as the clinical event. AUC, area under the curve.

*EGFR* T790M detection from liquid biopsy is not only a non-invasive testing method but also a strategy for monitoring the therapeutic efficacy of EGFR-TKIs. An ultra-sensitive method is required to achieve these aims, and we still needed to address the issue of determining the extent to which the amount of quantitative *EGFR* T790M corresponds to the clinical progress of the disease. The identification of an optimal cut-off value can alert practitioners to the imminent failure of treatment. Plasma samples from five patients (PR232, PR251, PR306, PR353, and PR366) underwent *EGFR* T790M testing by PNA-MALDI-TOF MS at various stages of treatment, including pre-treatment, two subsequent follow-ups, and at a point at PD (Fig 4A). To standardize the testing procedure, different volumes of plasma (2 ml, 1 ml, 0.5 ml, and 0.2 ml) were drawn for DNA extraction at each stage. The mutation frequency of T790M was plotted for each volume of plasma relative to the patient and time point (Fig 4A). Taking PD as the clinical event for ROC curve analysis, the area under the curve (AUC) of 2 ml, 1 ml, 0.5 ml, and 0.2 ml starting plasma volumes were 0.96, 0.72, 0.60, and 0.54, respectively (Fig 4B). The optimal cut-off value of enriched *EGFR* T790M mutation frequency of entire cfDNA was found to be 15% based on the 2 ml test. For the plasma samples taken at the follow-up time nearest to PD (imminent PD), we found AUCs of 0.82, 0.68, 0.55, and 0.48 for the 2 ml, 1 ml, 0.5 ml, and 0.2 ml samples respectively (Fig 4C). The optimal cut-off value of enriched *EGFR* T790M mutation frequency of entire cfDNA was 5% based on the 2 ml plasma samples tested.

### Correlation of *EGFR* T790M detection for rebiopsy tumor tissue and cfDNA

One hundred and three patients with first-line EGFR-TKIs treatment failure and rebiopsy tumor tissue available for testing were identified from TCVGH to evaluate the correlations in T790M detection (Tables 2 and S1 Table). The mean mutation frequency of *EGFR* T790M-positive cfDNA was above 15% in all cases (mean = 59.8%). The overall concordance of T790M in tissue biopsies and cfDNA samples was 78.6% (81/103) (S3 Table). PNA-MALDI-TOF MS showed that the sensitivity (positive percent agreement between tumor and cfDNA) of T790M was 67.4% (31/46) and specificity (negative percent agreement between tumor and cfDNA) was 87.7% (50/57). In addition, the positive prediction value (PPV) and negative prediction value (NPV) were 81.6% (31/38) and 76.9% (50/65), respectively.

### Clinical responsiveness of osimertinib by PNA-MALDI-TOF MS detection

Of the 103 patients to whom we applied methods of plasma EGFR T790M detection, 49 were receiving osimertinib and 46 of 49 were selected for further analysis, after excluding three patients who died within two weeks of osimertinib treatment or did not have a follow-up (Table 3). Among the evaluable responses we collected (n = 44), the ORR was 70.6% (24/34) for the tumor tested and 69.6% (16/23) for the cfDNA. For the T790M-negative patients, the ORR was 30.0% (3/10) when the tumors were tested and 52.4% (11/21) for the cfDNA.

**Table 3:** Comparison of tumor and cfDNA EGFR T790M and osimertinib treatment response.

	Tumor^1^	cfDNA^2^
*EGFR* T790M	103	103
positive	46 (44.7%)	38 (36.9%)
with osimertinib treatment	37 (80.4%)	24 (63.2%)
PR	24 (64.9%)	16 (66.7%)^3^
SD	8 (21.6%)	5 (20.8%)
PD	2 (5.4%)	2 (8.3%)
N/A^4^	1 (2.7%)	0 (0.0%)
Excluded^5^	2 (5.4%)	1 (4.2%)
without osimertinib treatment	9 (19.6%)	14 (36.8%)
negative	57 (55.3%)	65 (63.1%)
with osimertinib treatment	12 (21.1%)	25 (38.5%)
PR	3 (25.0%)^3^	11 (44.0%)
SD	3 (25.0%)	6 (24.0%)
PD	4 (33.3%)	4 (16.0%)
N/A^4^	1 (8.3%)	2 (8.0%)
Excluded^5^	1 (8.3%)	2 (8.0%)
without osimertinib treatment	45 (78.9%)	40 (61.5%)

PR, partial response; SD, stable disease; PD, progressive disease

^1^Tumor *EGFR* T790M was tested by MALDI-TOF MS.

^2^cfDNA *EGFR* T790M was tested by PNA-MALDI-TOF MS.

^3^One patient with PR response had *EGFR* T790M positive in cfDNA but negative in tumor.

^4^Patients with non-measurable lesion.

^5^Patients died within 2 weeks after osimertinib treatment or loss of follow-up.

To further evaluate the effectiveness of PNA-MALDI-TOF MS in cfDNA *EGFR* T790M detection, a parallel comparison between our approach and COBAS EGFR Mutation Test V2 (COBAS) was conducted. cfDNA from 20 patients receiving osimertinib treatment with PD or partial response from the third group were tested for *EGFR* T790M using both methods (Table 4). The analytical sensitivity of PNA-MALDI-TOF MS and COBAS was 61.5% (8/13) and 30.8% (4/13) when compared with results from tissue. The analytical specificity of PNA-MALDI-TOF MS and COBAS was 85.7% (6/7) and 85.7% (6/7) when compared with results from tissue. For clinical outcome correlation, among the six patients with PD, two had *EGFR* T790M by PNA-MALDI-TOF MS, while one by COBAS. Among 14 patients exhibiting partial response, six were shown to have *EGFR* T790M by PNA-MALDI-TOF MS while only three were revealed with COBAS. There were two invalid cases when COBAS was used, but none when PNA-MALDI-TOF MS was used (Table 4).

**Table 4:** Comparison of cfDNA T790M mutation detection by PNA-MALDI-TOF MS and COBAS.

Patient ID	Best Response to osimertinib	PFS (month)	*EGFR* T790M		
			Tissue	cfDNA by PNA MALDI-TOF MS	Plasma by COBAS
1	PD	1.3	-	-	-
2	PD	1.4	-	-	-
3	PD	1.7	-	-	-
4	PD	2.2	+	+	-
5	PD	3.0	-	-	-
6	PD	3.5	+	+	+
7	PR	6.3	+	-	-
8	PR	6.5	+	+	invalid
9	PR	7.0	+	-	invalid
10	PR	7.0	+	-	-
11	PR	9.3	+	+	+
12	PR	9.6	+	-	-
13	PR	10.0	+	-	-
14	PR	10.7	+	+	-
15	PR	10.8	+	+	-
16	PR	12.1	-	-	-
17	PR	13.5	-	-	-
18	PR	14.0	+	+	+
19	PR	14.6	-	+	+
20	PR	18.8	+	-	-

## Discussion

After the approval of osimertinib, testing for *EGFR* T790M became a standard technique in patient care, but the problem of rebiopsy availability has remained. cfDNA testing from liquid biopsy samples has emerged as a way of meeting clinical needs. In this study, we established an ultra-sensitive method that combined PNA with MALDI-TOF MS to identify *EGFR* T790M in cfDNA primarily taken from patients with EGFR-TKIs treatment failure. For MALDI-TOF MS, although we had applied it routinely in clinical practice, the sensitivity (1% mutant alleles frequency) for cfDNA mutations detection is insufficient. PNA has been widely utilized in genetic testing due to its propensity to improve detection sensitivity (detection limitation) through unbalanced PCR amplification. The major principle of PNA is mutant allele enrichment that can be suitable for specimens like plasma samples, which would otherwise show only trace amounts of mutant alleles [19–21]. PNA-MALDI-TOF MS can improve detection limitation from 1% to 0.1% (Fig 3), which allows the gathering of useful results from cfDNA testing. It should be noticed that T790M specific PNA only enriched T790M mutation frequency and did not affect the detection other EGFR mutations such as L858R (Fig 3).

Compared with other ultra-sensitive methods such as BEAMing [13, 22] as well as a PNA based QPCR method [23], our method had comparable analytical sensitivity (0.1% vs. 0.01–0.06% MAF). This may be caused by the added PNA interfered the background of MALDI-TOF MS or the design of PNA sequence. However, the strategy and the concept of PNA-MALDI-TOF MS for monitoring is feasible. The most important result of this study was the identification of the clinical *EGFR* T90M cut-off in cfDNA for PD and imminent PD. In clinical practice, this method allows practitioners to both identify the existence of mutations and the threat of PD when managing cancer. In the mention of method comparison, although PNA-MALDI-TOF MS exhibits higher testing successful rate compared with COBAS in a small number of samples (n = 20), a larger cohort for further validation is necessary. However, it should be noted some limitations and clarifications need to be mentioned before clinical practice. 1. Although the cut-offs from a very limited number of patients can be established to predict PD or imminent PD, a much larger cohort of patients should be further validated including comparisons between diagnostic methods. 2. The MAF cut-offs identified in this study was the consequence of enrichment by PNA. It should be “enriched” MAF and may not reflect the real frequency in cfDNA. 3. Although the MAF in cfDNA seems to correlate with clinical outcome [24, 25], it is worthy to further address whether the prediction power of cut-off value will be improved after adjusting the fluctuation of total cfDNA in each patient. 4. Among 5 patients in the training cohort, 2 patients (PR306 and PR251) were not fully correlated with imminent PD. This may be caused by dynamic and variable cfDNA in each patient. However, it can be observed that the T790M MAF of these 2 patients were relative higher than other patients during treatment. This phenomenon can be alerted and intensively monitored in the future. Furthermore, compared with widely used COBAS or ddPCR based platforms, translational research-based lab developed test such as MALDI-TOF MS in this study should be methodologically and clinically validated prior to clinical practice.

Based on a meta-analysis and systematic review, the diagnostic accuracy of cfDNA for *EGFR*-mutations ranges considerably [26]. It is very difficult to fairly compare each study in parallel because of many confounding factors such as the condition of the plasma samples, nucleic acid extraction, detection variation, and heterogeneity of patients. In terms of the comparison of *EGFR* T790M testing using cfDNA and tumor tissue, our method demonstrated a 78.6% concordance, which is above the average in previous studies comparing several methods [22, 27]. It is important to consider that the inconsistent results between the tissue and cfDNA may be clinically significant. For example, recent reports have indicated that *EGFR* T790M may be identified in cfDNA but missing in tumor biopsy samples [28]. This suggests that cfDNA tests may be useful for patients with actionable mutations missed by conventional tumor biopsy analysis due to intratumoral heterogeneity [29, 30]. Therefore, the comparison of mutation results between from tumors and from cfDNA may only mention about analytical sensitivity. The correlation between results and clinical responses and outcomes needs to be further understood for treatment decisions and managements.

Many methods have been developed for *EGFR*-mutation detection in cfDNA. For example, the well-established but unquantified method of the Scorpion ARMS-based *EGFR* detection platform has demonstrated sensitivity and specificity levels of 67.5% and 99.8%, respectively [31]. Furthermore, ddPCR is a widely used and quantifiable plasma mutation detection method with ultra-sensitivity. Although the best reported data for patients with lung adenocarcinoma were obtained with an *EGFR*-mutation test that uses ddPCR, which resulted in a 92% sensitivity and 100% specificity, lower or more varied levels of sensitivity (29.0–100.0%) and specificity (50.0–100.0%) have also been observed in *EGFR* T790M testing [22, 26, 30, 32, 33]. Compared with the recent phase II clinical trial report, our method showed better statistical analysis values, such as in positive and negative percent agreement [34]. Therefore, PNA-MALDI-TOF MS can be a useful alternative method to facilitate quantified mutation detection in cfDNA. In terms of clinical outcome analysis, our results indicated that T790M-positive plasma had 69.6% ORR, similar to that from T790M-positive tumors (70.6% ORR) (Table 3). This is consistent with and even surpasses the predicted response value in previous studies [26, 30]. In contrast, of 65 patients with T790M-negative plasma in this study, 15 were tumor positive (false negative = 23.1%), which is lower than another study [13]. Therefore, the value in testing tumor *EGFR* T790M in negative plasma patients should be recognized. On the other hand, of 38 patients with T790M-positive plasma, seven had T790M-negative tumors (false positive = 18.4%), suggesting that clonal heterogeneity of tumors may contribute to this result. Among these seven patients with T790M-negative tumors, one of them received osimertinib and had a partial response. This suggests that plasma testing could be considered for evaluating the prospective benefit of osimertinib treatment for a particular patient.

The concept of dynamic mutational monitoring to determine the tendency of relapse in patients should be put into routine practice. However, to determine the amount of resistant mutations sufficiently confer to PD is a growing issue. This study mentioned the dynamic change of *EGFR* T790M in cfDNA and the cut-off value during PD. Undoubtedly, the development of methodologies for mutation detection in cfDNA requires continued improvement to meet clinical needs. For each method developed, we recommend a standardized testing procedure and an emphasis on clinical interpretations. Based on this particular study, 60 μl eluted DNA extracted from 2 ml of patient plasma is recommended for biochemical reaction. Two important findings were confirmed in this study. First, we have shown that PNA-MALDI-TOF MS can be used efficiently for *EGFR* T790M testing in cfDNA with accuracy and flexibility. Although the sensitivity of the method is the central consideration, clinical utility of test results for patients undergoing treatment should be considered as well. Secondly, we have shown that in terms of operation procedure, turnaround time, and especially cost, PNA-MALDI-TOF MS is an alternative cfDNA detection method. Of course, it should be noticed that the amount of cfDNA in plasma sample differs from patient to patient. The cut-off value identification may be affected due to variations of the T790M MAF no matter which method used. A large prospective cohort to validate the T790M mutation frequency for clinical utilities is required. Finally, although MALDI-TOF MS has not widely used like COBAS or ddPCR in most hospitals for clinical practice, this study highlights its advantages in applications of lab developed multiplex customized cfDNA mutational monitoring and its flexibility for clinical unmet needs.

## Supporting information

S1 Table Comparison of tumor and cfDNA *EGFR* T790M and osimertinib treatment response. (DOCX)

S2 Table Precision analysis of EGFR T790M MAF by PNA-MALDI-TOF MS in triplicates. (DOCX)

S3 Table Statistics of *EGFR* T790M test in tumor and cfDNA. (DOCX)

S1 Fig Precision evaluation of PNA-MALDI-TOF MS. EGFR T790M harbored H1975 cells was utilized for between-run reproducibility and within-run repeatability testing. Each testing contained 20 duplicates and coefficient of variation was indicated.
(TIF)
