# Supplementary material for: Mutational monitoring of EGFR T790M in cfDNA for clinical outcome prediction in EGFR-mutant lung adenocarcinoma
Source: PLoS One. 2018 Nov 16;13(11):e0207001. doi: 10.1371/journal.pone.0207001 (PMC6239293; doi:10.1371/journal.pone.0207001)
Supplement: S1 Table — (DOCX) [file pone.0207001.s001.docx]

| **S1 Table.** Comparison of tumor and cfDNA *EGFR* T790M and Osimertinib treatment response. | | | | |
| --- | --- | --- | --- | --- |
| Patient ID | EGFR T790M in tumor^1^ | EGFR T790M in cfDNA^2^ | Osimertinib treatment | Osimertinib response |
| 1 | + | + | + | PD |
| 2 | + | - | + | N/A^3^ |
| 3 | + | + | + | SD |
| 4 | - | - | - | N/A |
| 5 | - | - | - | N/A |
| 6 | - | - | - | N/A |
| 7 | + | + | - | N/A |
| 8 | + | + | + | PR |
| 9 | - | - | - | N/A |
| 10 | - | + | - | N/A |
| 11 | - | - | - | N/A |
| 12 | - | - | - | N/A |
| 13 | - | - | - | N/A |
| 14 | - | + | - | N/A |
| 15 | - | - | + | PR |
| 16 | - | - | - | N/A |
| 17 | + | + | + | PR |
| 18 | + | - | + | PR |
| 19 | - | - | + | PD |
| 20 | + | - | + | N/A |
| 21 | + | + | + | PR |
| 22 | - | - | - | N/A |
| 23 | - | - | + | PD |
| 24 | + | - | + | PR |
| 25 | - | - | - | N/A |
| 26 | + | + | - | N/A |
| 27 | + | + | + | PR |
| 28 | - | - | + | SD |
| 29 | - | - | - | N/A |
| 30 | - | - | - | N/A |
| 31 | - | - | - | N/A |
| 32 | - | + | - | N/A |
| 33 | + | + | - | N/A |
| 34 | - | - | - | N/A |
| 35 | - | - | - | N/A |
| 36 | + | + | + | PR |
| 37 | + | + | + | PR |
| 38 | - | - | + | PD |
| 39 | + | - | + | PR |
| 40 | - | - | - | N/A |
| 41 | + | - | + | PR |
| 42 | + | + | + | PR |
| 43 | + | + | - | N/A |
| 44 | - | + | - | N/A |
| 45 | - | - | - | N/A |
| 46 | + | + | - | N/A |
| 47 | - | - | - | N/A |
| 48 | - | - | - | N/A |
| 49 | + | - | + | PR |
| 50 | - | - | - | N/A |
| 51 | + | + | + | PR |
| 52 | + | - | + | SD |
| 53 | + | + | + | N/A^3^ |
| 54 | - | - | - | N/A |
| 55 | - | + | - | N/A |
| 56 | + | + | + | PR |
| 57 | - | + | + | PR |
| 58 | - | - | + | N/A^4^ |
| 59 | + | + | + | SD |
| 60 | - | - | - | N/A |
| 61 | + | + | + | PR |
| 62 | + | + | + | PR |
| 63 | + | + | + | PR |
| 64 | - | - | - | N/A |
| 65 | - | - | - | N/A |
| 66 | + | - | + | PR |
| 67 | + | + | + | SD |
| 68 | + | + | + | SD |
| 69 | + | + | + | SD |
| 70 | + | + | + | PR |
| 71 | + | + | + | PR |
| 72 | + | + | - | N/A |
| 73 | + | + | + | PD |
| 74 | + | - | + | PR |
| 75 | + | - | + | SD |
| 76 | + | - | + | SD |
| 77 | - | - | + | PR |
| 78 | - | - | - | N/A |
| 79 | - | - | - | N/A |
| 80 | - | - | - | N/A |
| 81 | - | - | - | N/A |
| 82 | - | - | - | N/A |
| 83 | - | - | - | N/A |
| 84 | - | - | - | N/A |
| 85 | - | - | - | N/A |
| 86 | - | - | - | N/A |
| 87 | + | + | - | N/A |
| 88 | - | - | - | N/A |
| 89 | - | - | - | N/A |
| 90 | - | - | + | SD |
| 91 | - | - | - | N/A |
| 92 | - | - | + | SD |
| 93 | - | - | - | N/A |
| 94 | + | + | - | N/A |
| 95 | - | - | - | N/A |
| 96 | - | + | - | N/A |
| 97 | - | - | + | N/A |
| 98 | - | - | + | PD |
| 99 | - | - | - | N/A |
| 100 | + | - | + | PR |
| 101 | + | - | + | PR |
| 102 | + | - | - | N/A |
| 103 | + | + | + | PR |

PR, partial response; SD, stable disease; PD, progressive disease

^1^Tumor *EGFR* T790M was test by MALDI-TOF MS.

^2^cfDNA *EGFR* T790M was test by PNA-MALDI-TOF MS.

^3^Died after osimertinib treatment <2 weeks.

^4^Loss of follow-up
