# Supplementary material for: Mutational monitoring of EGFR T790M in cfDNA for clinical outcome prediction in EGFR-mutant lung adenocarcinoma
Source: PLoS One. 2018 Nov 16;13(11):e0207001. doi: 10.1371/journal.pone.0207001 (PMC6239293; doi:10.1371/journal.pone.0207001)
Supplement: S2 Table — (DOCX) [file pone.0207001.s002.docx]

| **S2 Table.** Precision analysis of EGFR T790M MAF by PNA-MALDI-TOF MS in triplicates. | | | | | | | |
| --- | --- | --- | --- | --- | --- | --- | --- |
|  | Sample ID | 1st | 2nd | 3rd | Mean | SD | CV(%) |
| Negative |  |  |  |  |  |  |  |
|  | 001 | 0.0 | 0.0 | 0.0 | 0.0 | 0.0 | NA |
|  | 002 | 0.0 | 0.0 | 0.0 | 0.0 | 0.0 | NA |
|  | 003 | 0.0 | 0.0 | 0.0 | 0.0 | 0.0 | NA |
|  | 004 | 0.0 | 0.0 | 0.0 | 0.0 | 0.0 | NA |
|  | 005 | 0.0 | 0.0 | 0.0 | 0.0 | 0.0 | NA |
| Low MAF (<5%) |  |  |  |  |  |  |  |
|  | 006 | 2.9 | 1.5 | 2.3 | 2.2 | 0.7 | 31.7 |
|  | 007 | 1.3 | 1.7 | 1.6 | 1.5 | 0.2 | 12.8 |
|  | 008 | 2.7 | 2.3 | 2.5 | 2.5 | 0.2 | 8.2 |
|  | 009 | 2.7 | 2.0 | 2.6 | 2.4 | 0.3 | 14.1 |
|  | 010 | 2.4 | 1.5 | 1.9 | 2.0 | 0.5 | 22.9 |
| Median MAF (5-15%) |  |  |  |  |  |  |  |
|  | 011 | 14.9 | 14.8 | 14.6 | 14.7 | 0.1 | 0.9 |
|  | 012 | 6.7 | 3.5 | 5.3 | 5.2 | 1.6 | 31.0 |
|  | 013 | 12.8 | 8.7 | 9.3 | 10.3 | 2.2 | 21.3 |
|  | 014 | 7.8 | 7.7 | 7.6 | 7.7 | 0.1 | 1.0 |
|  | 015 | 10.1 | 12.5 | 11.5 | 11.4 | 1.2 | 10.4 |
| High MAF (>15%) |  |  |  |  |  |  |  |
|  | 016 | 25.9 | 27.9 | 26.1 | 26.6 | 1.1 | 4.1 |
|  | 017 | 68.2 | 69.5 | 68.5 | 68.7 | 0.7 | 1.0 |
|  | 018 | 37.3 | 48.0 | 44.3 | 43.2 | 5.4 | 12.5 |
|  | 019 | 98.8 | 100.0 | 99.1 | 99.3 | 0.6 | 0.6 |
|  | 020 | 100.0 | 100.0 | 100.0 | 100.0 | 0.0 | 0.0 |

MAF, mutation allele frequency.
