# Supplementary material for: Mutational monitoring of EGFR T790M in cfDNA for clinical outcome prediction in EGFR-mutant lung adenocarcinoma
Source: PLoS One. 2018 Nov 16;13(11):e0207001. doi: 10.1371/journal.pone.0207001 (PMC6239293; doi:10.1371/journal.pone.0207001)
Supplement: S3 Table — (DOCX) [file pone.0207001.s003.docx]

| **S3 Table.** Statistics of *EGFR* T790M test in tumor and cfDNA. | | | |
| --- | --- | --- | --- |
|  |  | Tissue *EGFR* T790M | |
|  |  | + | - |
| cfDNA *EGFR* T790M | + | 31 | 7 |
|  | - | 15 | 50 |

sensitivity= 31/46=67.4%

specificity = 50/57=87.7%

PPV = 31/38=81.6%

NPV = 50/65=76.9%
