# Supplementary figures and images for: Mutational monitoring of EGFR T790M in cfDNA for clinical outcome prediction in EGFR-mutant lung adenocarcinoma
Source: PLoS One. 2018 Nov 16;13(11):e0207001. doi: 10.1371/journal.pone.0207001 (PMC6239293; doi:10.1371/journal.pone.0207001)

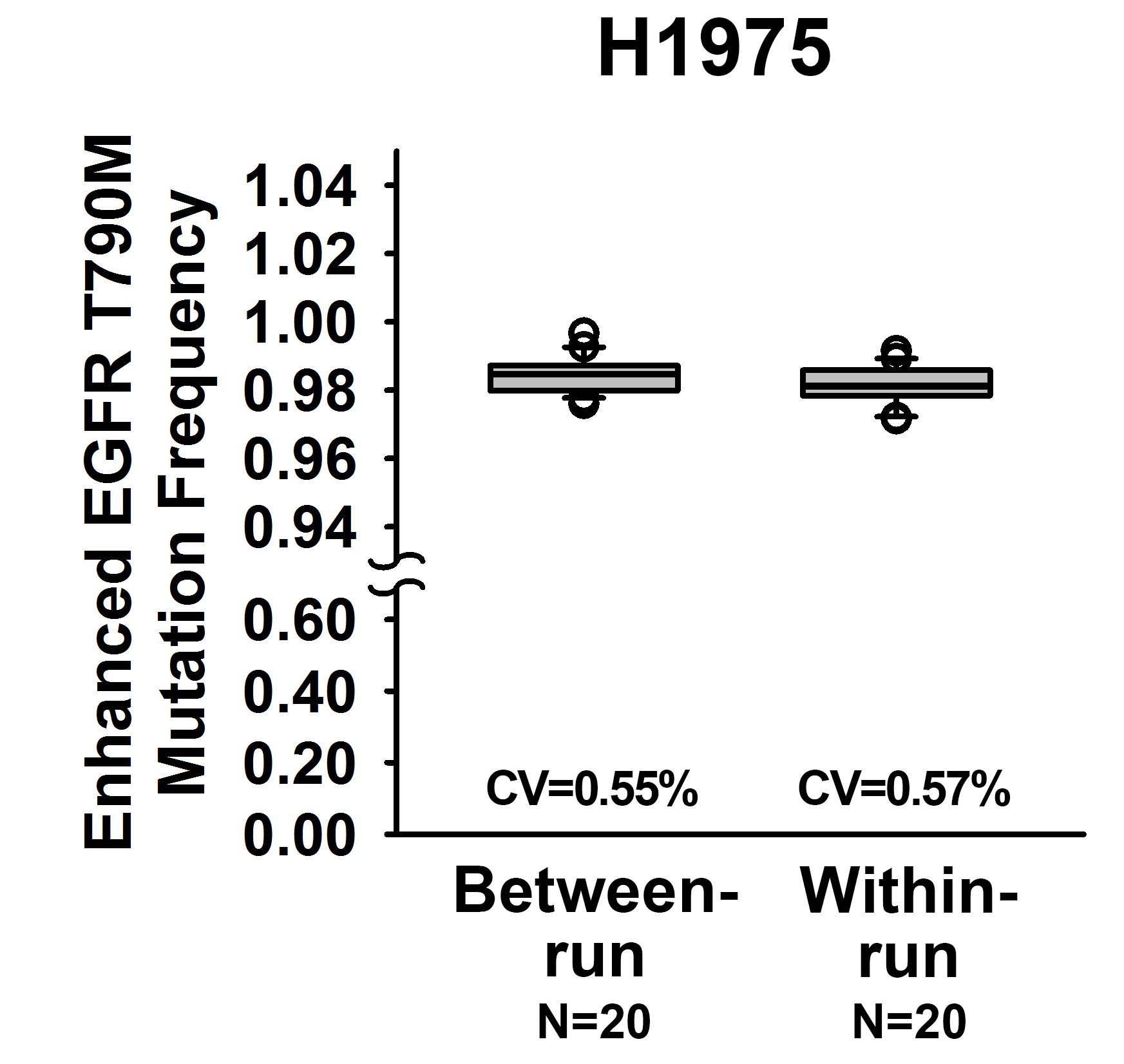

Supplement: S1 Fig — EGFR T790M harbored H1975 cells was utilized for between-run reproducibility and within-run repeatability testing. Each testing contained 20 duplicates and coefficient of variation was indicated. (TIF) [file pone.0207001.s004.tif]
